# Hypoxia-Mediated Mechanism of MUC5AC Production in Human Nasal Epithelia and Its Implication in Rhinosinusitis

**DOI:** 10.1371/journal.pone.0098136

**Published:** 2014-05-19

**Authors:** Yoon-Ju Kim, Hyung-Ju Cho, Woo-Chul Shin, Hyun-Ah Song, Joo-Heon Yoon, Chang-Hoon Kim

**Affiliations:** 1 Department of Otorhinolaryngology, Yonsei University College of Medicine, Seoul, Korea; 2 The Airway Mucus Institute, Yonsei University College of Medicine, Seoul, Korea; 3 BK 21 Project for Medical Science, Yonsei University College of Medicine, Seoul, Korea; 4 Research Center for Human Natural Defense System, Yonsei University College of Medicine, Seoul, Korea; 5 Hana ENT Hospital, Seoul, Korea; University of Cologne, Germany

## Abstract

**Background:**

Excessive mucus production is typical in various upper airway diseases. In sinusitis, the expression of MUC5AC, a major respiratory mucin gene, increases. However, the mechanisms leading to mucus hypersecretion in sinusitis have not been characterized. Hypoxia due to occlusion of the sinus ostium is one of the major pathologic mechanisms of sinusitis, but there have been no reports regarding the mechanism of hypoxia-induced mucus hypersecretion.

**Methods and Findings:**

This study aims to identify whether hypoxia may induce mucus hypersecretion and elucidate its mechanism. Normal human nasal epithelial (NHNE) cells and human lung mucoepidermoid carcinoma cell line (NCI-H292) were used. Sinus mucosa from patients was also tested. Anoxic condition was in an anaerobic chamber with a 95% N_2_/5% CO_2_ atmosphere. The regulatory mechanism of MUC5AC by anoxia was investigated using RT-PCR, real-time PCR, western blot, ChIP, electrophoretic mobility shift, and luciferase assay. We show that levels of MUC5AC mRNA and the corresponding secreted protein increase in anoxic cultured NHNE cells. The major transcription factor for hypoxia-related signaling, HIF-1α, is induced during hypoxia, and transfection of a mammalian expression vector encoding HIF-1α results in increased MUC5AC mRNA levels under normoxic conditions. Moreover, hypoxia-induced expression of MUC5AC mRNA is down-regulated by transfected HIF-1α siRNA. We found increased MUC5AC promoter activity under anoxic conditions, as indicated by a luciferase reporter assay, and mutation of the putative hypoxia-response element in MUC5AC promoter attenuated this activity. Binding of over-expressed HIF-1α to the hypoxia-response element in the MUC5AC promoter was confirmed. In human sinusitis mucosa, which is supposed to be hypoxic, expression of MUC5AC and HIF-1α is higher than in control mucosa.

**Conclusion:**

The results indicate that anoxia up-regulates MUC5AC by the HIF-1α signaling pathway in human nasal epithelia and suggest that hypoxia might be a pathogenic mechanism of mucus hypersecretion in sinusitis.

## Introduction

The epithelial surface of mammalian respiratory, gastrointestinal, and reproductive tracts is coated by mucus, which is a mixture of water, glycoproteins, proteins, and lipids. Mucus plays a role in the innate immune system in epithelial tissues by providing a protective barrier against pathogens or toxins. Mucin is the major molecular constituent of mucus and has been implicated in numerous airway diseases. There are 20 human genes deposited in GenBank that encode secretory or membrane-tethered mucin [1]. Among them, MUC5AC and MUC5B, two major secretory mucins found in the airway, are over-produced in chronic rhinosinusitis [2]. In the sinus, MUC5AC is predominant, relative to MUC5B, because it is commonly secreted from epithelial cells but not from the submucosal gland [3]. On the other hand, MUC5B is secreted mainly from the submucosal gland, and the gland density in the sinus is lower than in the nasal cavity [4].

Expression of MUC5AC is controlled by various mediators, such as interleukin (IL)-1β [5], IL-6 [6], IL-13 [7], IL-17 [6], or tumor necrosis factor-α. In human sinusitis, reduced oxygen tension in the sinus is considered as a major pathophysiology, and its severity is related to the oxygen level of the sinus [8]. Hypoxia can result in the failure of transepithelial oxygenation, nonvascularized exudates, or the tendency of inflammatory hyperplasia to exceed neovascularization [9]. However, the relationship between MUC5AC and hypoxia has been little investigated.

In hypoxic conditions, the activity of hypoxia-inducible factor 1 (HIF-1) is very important for maintaining oxygen homeostasis by transcriptional activation of erythropoietin [10], vascular endothelial growth factor [11], heme oxygenase-1 [12], and transferrin [13]. The structure of HIF-1 is a heterodimer composed of α and β subunits, and it belongs to the PER-ARNT-SIM subfamily of the basic helix-loop-helix family [14]. The activity of HIF-1 is primarily determined by hypoxia-induced stabilization of HIF-1α, which becomes rapidly degraded through the ubiquitin-proteasome pathway [15]. Under hypoxic conditions, stable HIF-1α dimerizes with HIF-1β and binds to the hypoxia-response element (HRE) to recruit the transcription coactivator p300/CBP onto the promoter of hypoxia-responsive genes for transcriptional activation [15]–[17]. The HRE (approximately 100 bp in size) is usually located at the proximal promoter, and it contains one or more HIF-1 binding sites (consensus sequence 5′-[A/G]CGTG-3′) [18]. The presence of the HRE region is critical because its mutation inactivates the transcriptional response to hypoxia [19], [20]. We have noticed that the promoter region of the MUC5AC gene, studied by Li D. et al. [21] or Young HW et al. [22], contains a sequence that is very similar to the HRE, located within a 70-bp region upstream of the transcriptional start site. In this study, we have aimed to characterize this sequence as a functionally active HRE and gain greater understanding of the mechanism of hypoxia-induced MUC5AC gene regulation in nasal epithelium.

## Results

### Anoxia in NHNE cells induces the expression of MUC5AC mRNA and secretion of MUC5AC protein

**Figure 1:**
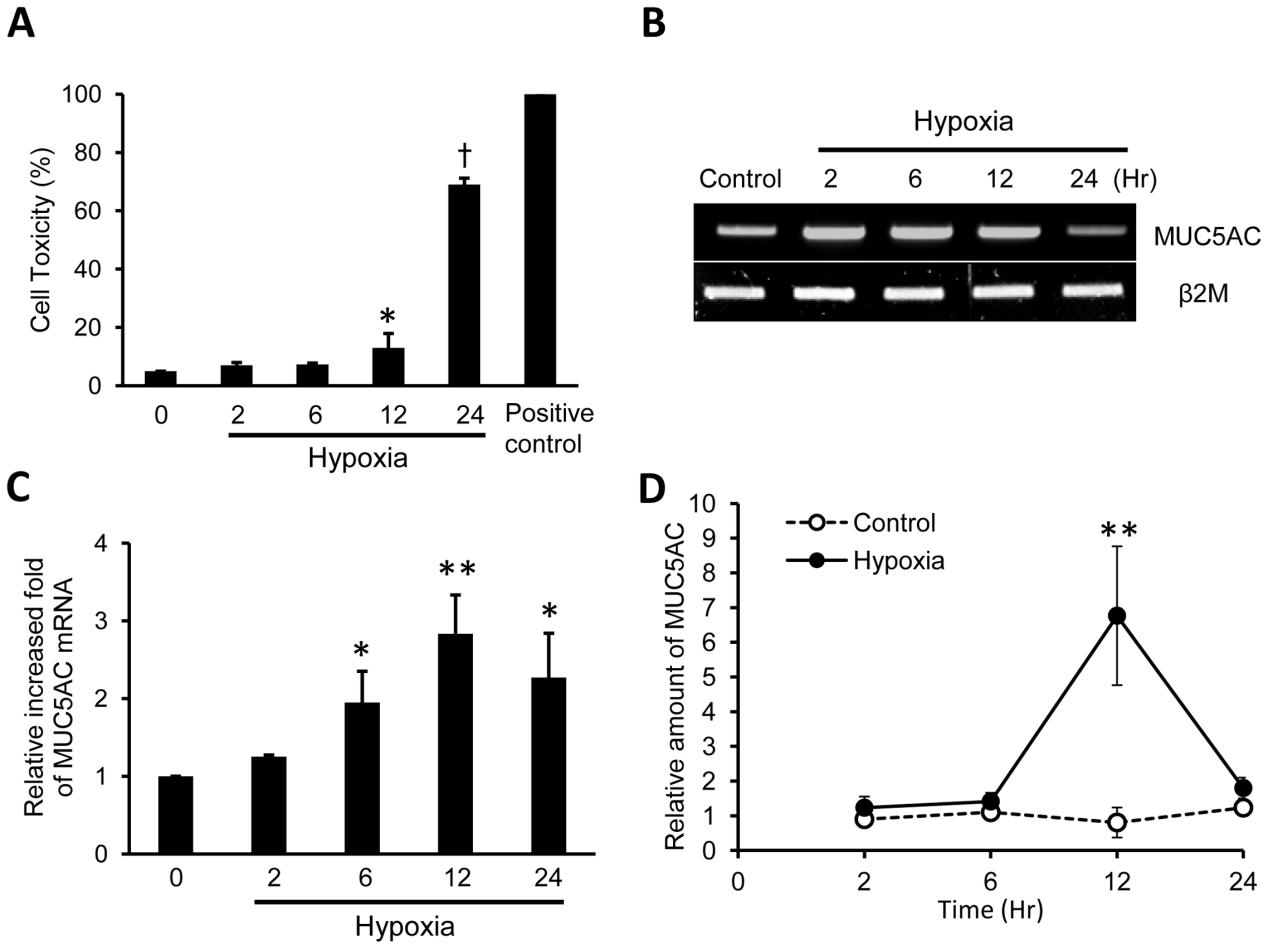
Anoxia induces the expression of mRNA and production of MUC5AC in NHNE cells. (**A**) MTT assay to determine cytotoxic effects of anoxia. Cell viability was not significantly affected by less than 6 h of anoxia (n = 3, *p<0.05, †p<0.0001). (**B**) The expression of the MUC5AC gene according to anoxia stimulation (95% N_2_/5% CO_2_ atmosphere in the hypoxic chamber) for 0–24 h in NHNE cells. MUC5AC gene expression was increased under anoxic conditions, measured by RT-PCR. (**C**) The expression of MUC5AC mRNA was maximally induced at 12 h during anoxia (measured by real-time PCR, n = 3, *p<0.05, **p<0.001). d) Mucus secretion was sampled at 2, 6, 12, and 24 h after incubating of NHNE cells under anoxic conditions and measured in dot blots with anti-MUC5AC antibody. Secreted MUC5AC was highest at 12 h under anoxic conditions (n = 3, **P<0.001). Data is shown as mean ± standard deviation.

Effects of anoxia on cell viability were tested by MTT assay (Fig. 1A), and it started to increase from 12 h (13%, p<0.05) and it was maximal at 24 h (69%, p<0.0001). To determine whether hypoxia affects MUC5AC gene expression, NHNE cells were incubated in the hypoxic chamber (95% N_2_/5% CO_2_), and MUC5AC mRNA was measured by RT-PCR (Fig. 1B) and real-time PCR (Fig. 1C) with increasing time, at 0, 2, 6, 12 and 24 h of anoxic treatment. Compared with normoxic conditions, levels of MUC5AC mRNA were increased as a function of time by anoxia from 6 h (p<0.05), with maximal induction at 12 h (p<0.001). To determine the secretion of MUC5AC protein, NHNE cells were incubated in the same manner, and the cellular apical secretion was analyzed by dot blot with anti-MUC5AC antibody. MUC5AC secretion was also increased by anoxia, and it was maximally increased at 12 h (two-way ANOVA, p<0.001) and decreased at 24 h (Fig. 1D).

### Anoxia induces HIF-1α protein levels in NHNE cells

**Figure 2:**
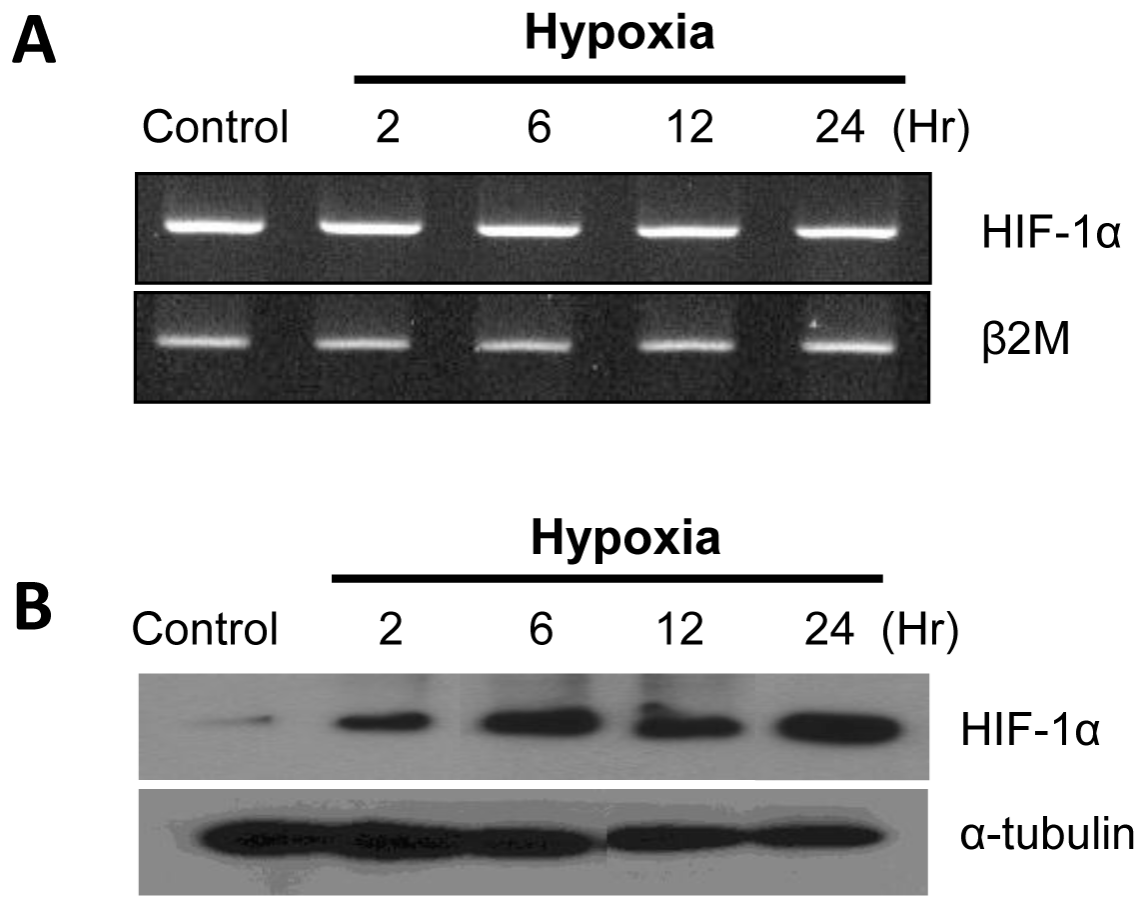
Anoxia induces HIF-1α protein levels in NHNE cells. The expression of HIF-1α mRNA and protein in NHNE cells under anoxia stimulation for 0–24 h. (**A**) The transcription of HIF-1α was not altered by anoxia, measured by RT-PCR. (**B**) Expression of HIF-1α protein was increased under anoxic conditions compared to the normoxic control (western blot).

To test whether hypoxia induces the expression of HIF-1α as a function of time, NHNE cells were incubated for 2, 6, 12, or 24 h under anoxic conditions. In these experiments, it may be seen that HIF-1α mRNA was not influenced by anoxia (Fig. 2A). Interestingly, however, anoxia increased the level of HIF-1α protein compared to the normoxic control cells (Fig. 2B).

### Gain- or loss-of-function study: Cellular over-expression versus inhibition of HIF-1α

**Figure 3:**
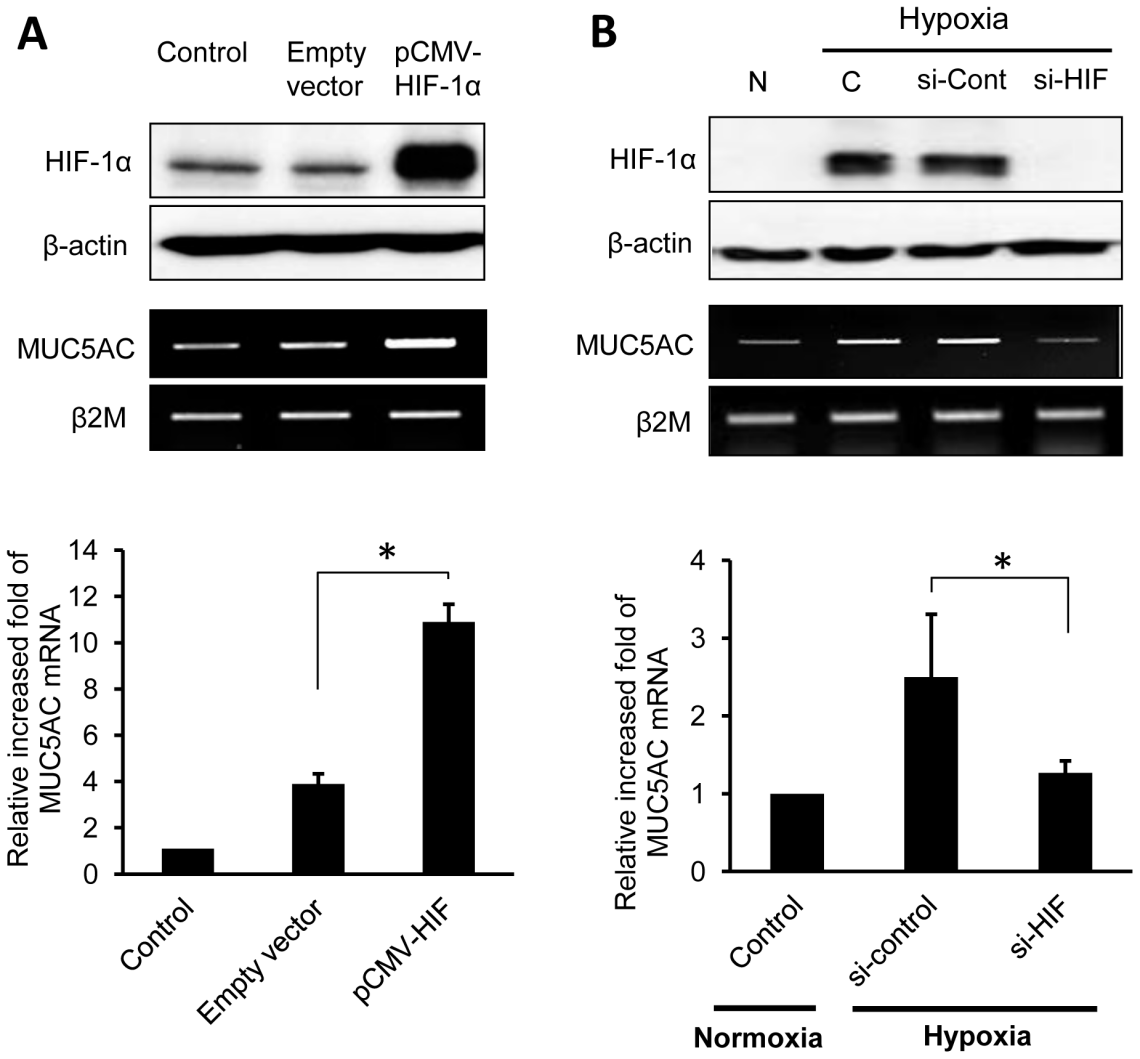
HIF-1α is involved in the regulation of MUC5AC expression. When HIF-1α expression is increased, MUC5AC expression is induced; conversely, when HIF-1α expression is decreased, MUC5AC expression is reduced accordingly. (**A**) Gain-of-function study with mammalian HIF-1α expression vector in NCI-H292 cells. DNA of the pCMV vector encoding HIF-1α, or empty pCMV vector without HIF-1α, was transiently transfected. The expression of both HIF-1α and MUC5AC gene was increased when transfected with pCMV-HIF-1α vector, compared to transfection of empty control vectors under normoxic conditions (n = 3, *p<0.05). (**B**) Loss-of-function study with transfected HIF-1α siRNA in NCI-H292 cells. The expression of both HIF-1α and MUC5AC gene was suppressed when transfected with siRNA of HIF-1α, compared to transfection of siRNA negative control under hypoxic conditions for 6 h (n = 3, *p<0.05). Data is shown as mean ± standard deviation. *(N; normoxia, C; control)*.

To determine whether HIF-1α is involved in the regulation of MUC5AC, a mammalian expression vector containing HIF-1α (pCMV-HIF-1α) (generously given by Dr. Koh EM, SMC, Seoul, Korea) was transfected into NCI-H292 cells for transient expression studies. Initially, western blot analysis showed increased HIF-1α expression in the cells that had been transfected with HIF-1α expression plasmids compared to the cells with empty vectors (Fig. 3A). Levels of MUC5AC mRNA, measured by RT-PCR and real-time PCR, indicated that transfection of plasmids encoding HIF-1α increased MUC5AC mRNA even under normoxic conditions (Fig. 3A).

To confirm the role of HIF-1α in MUC5AC expression, the HIF-1α gene was subjected to knock down by transfected small interfereing-RNA (si-RNA). The expression of HIF-1α was suppressed by HIF-1α siRNA, and MUC5AC mRNA was significantly reduced accordingly when compared to control-RNA transfection under anoxic conditions (Fig. 3B).

### Identification of hypoxia-responsive regions within the MUC5AC promoter

**Figure 4:**
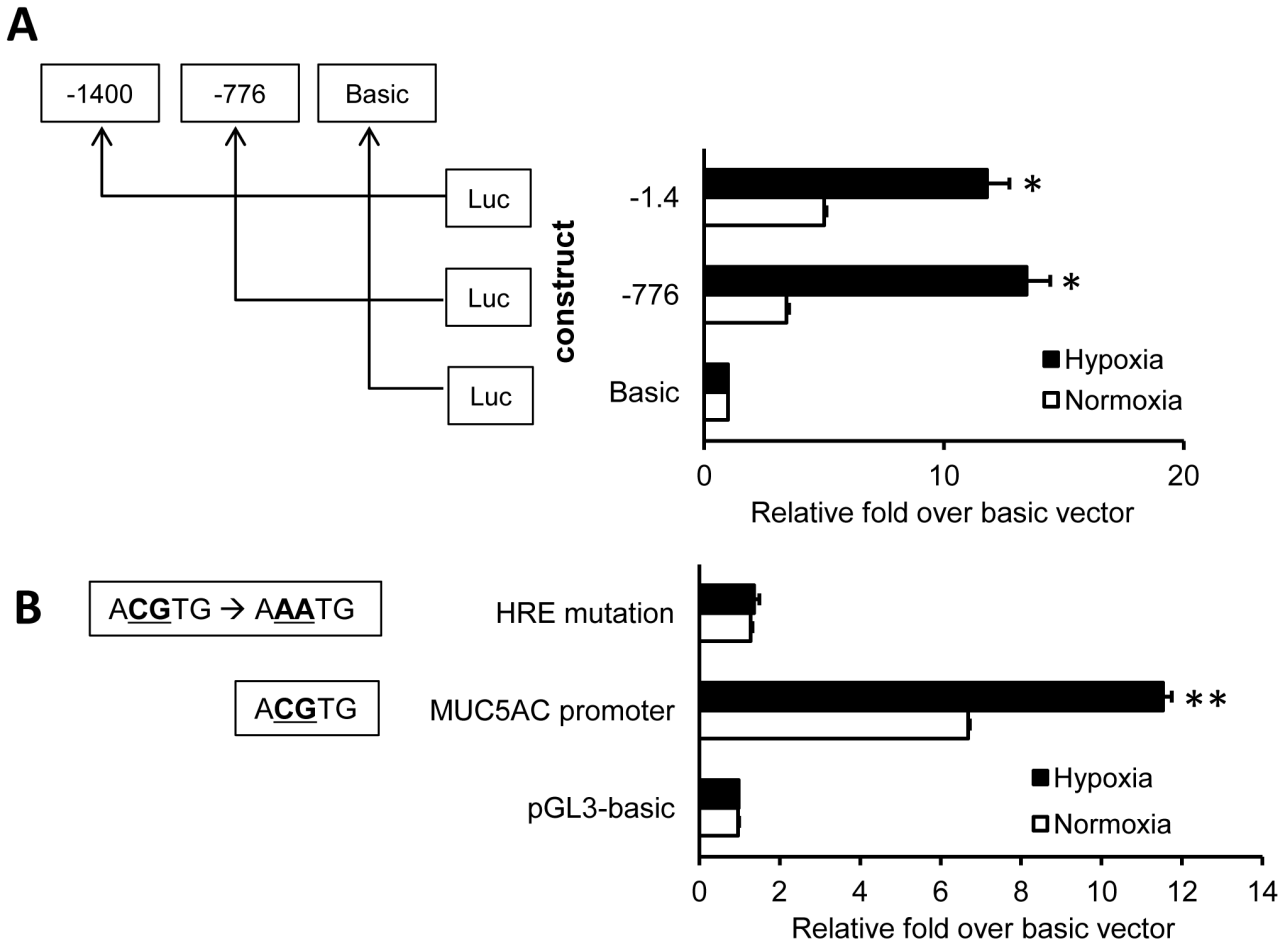
Luciferase reporter assay dependent on MUC5AC promoter sequences. (**A**) NCI-H292 cells were transiently transfected with the various deletion mutants and treated under normoxic or anoxic conditions for 6 h. Hypoxia selectively increased luciferase activity driven by sequences corresponding to the −1400/+4 and −776/+4 regions of the MUC5AC promoter, indicating that the −776/+4 region of MUC5AC promoter has a role in the cellular response to anoxia (n = 3, *P<0.01). (**B**) NCI-H292 cells were transfected with pGL3-basic vectors, pGL3 vectors containing the putative MUC5AC promoter, or pGL3 vectors containing the HRE-mutated MUC5AC promoter. After cells were incubated in the anoxic chamber for 6 h, luciferase activities were measured. Under anoxia conditions, the luciferase reporter activity of the wild-type MUC5AC promoter was increased by 8.3-fold compared to the HRE-mutated MUC5AC promoter, and by 11.2-fold compared to the pGL3-basic vectors (n = 3, **p<0.001). Data is shown as mean ± standard deviation.

Cells that had been transiently transfected with the various deletion mutants were treated by normoxia versus anoxia for 6 h. As shown in Fig. 4A, anoxia selectively increased reporter luciferase activity driven by two regions of the MUC5AC promoter (p<0.01), from positions −1400/+4 and −776/+4 (relative to the transcriptional start site). No effect was observed on fragments covering basic regions, indicating that the −776/+4 region of the MUC5AC promoter might be required for a response to anoxia. The HRE is located between 61–65 bp upstream of the MUC5AC gene, and its sequence is 5′-ACGTG-3′. To further investigate whether activation of the HRE is required for hypoxia-induced MUC5AC transcription, reporter luciferase assays that tested mutations in the wild-type HRE (5′-ACGTG-3′ mutated to 5′-AAATG-3′) were carried out (Fig. 4B). These wild-type HRE-mutated MUC5AC promoters were inserted into the pGL3 plasmid, which has a luciferase reporter gene. Cultures of NCI-H292 cells were transfected with pGL3 vectors containing putative MUC5AC promoter, pGL3 vectors with HRE-mutated MUC5AC promoter, and pGL3-basic vectors. In anoxia, the relative luciferase activity with pGL3-putative MUC5AC promoter was increased 8.3-fold compared to the HRE-mutated group (p<0.0001), and was increased 11.2-fold relative to the pGL3-basic vector group. In normoxia, the luciferase activity with plasmids encoding wild-type MUC5AC promoter was increased compared to the groups with either HRE-mutated promoter (p<0.0001) or pGL3-basic vector. Compared to expression under normoxic conditions, the luciferase activity was increased 1.7-fold in the cells with putative MUC5AC promoter under anoxic conditions (p<0.001). Anoxic stimulation did not increase the activity of luciferase reporter when the HRE of the MUC5AC promoter was mutated (p = 0.578).

### HRE is required for anoxia-induced MUC5AC transcription

**Figure 5:**
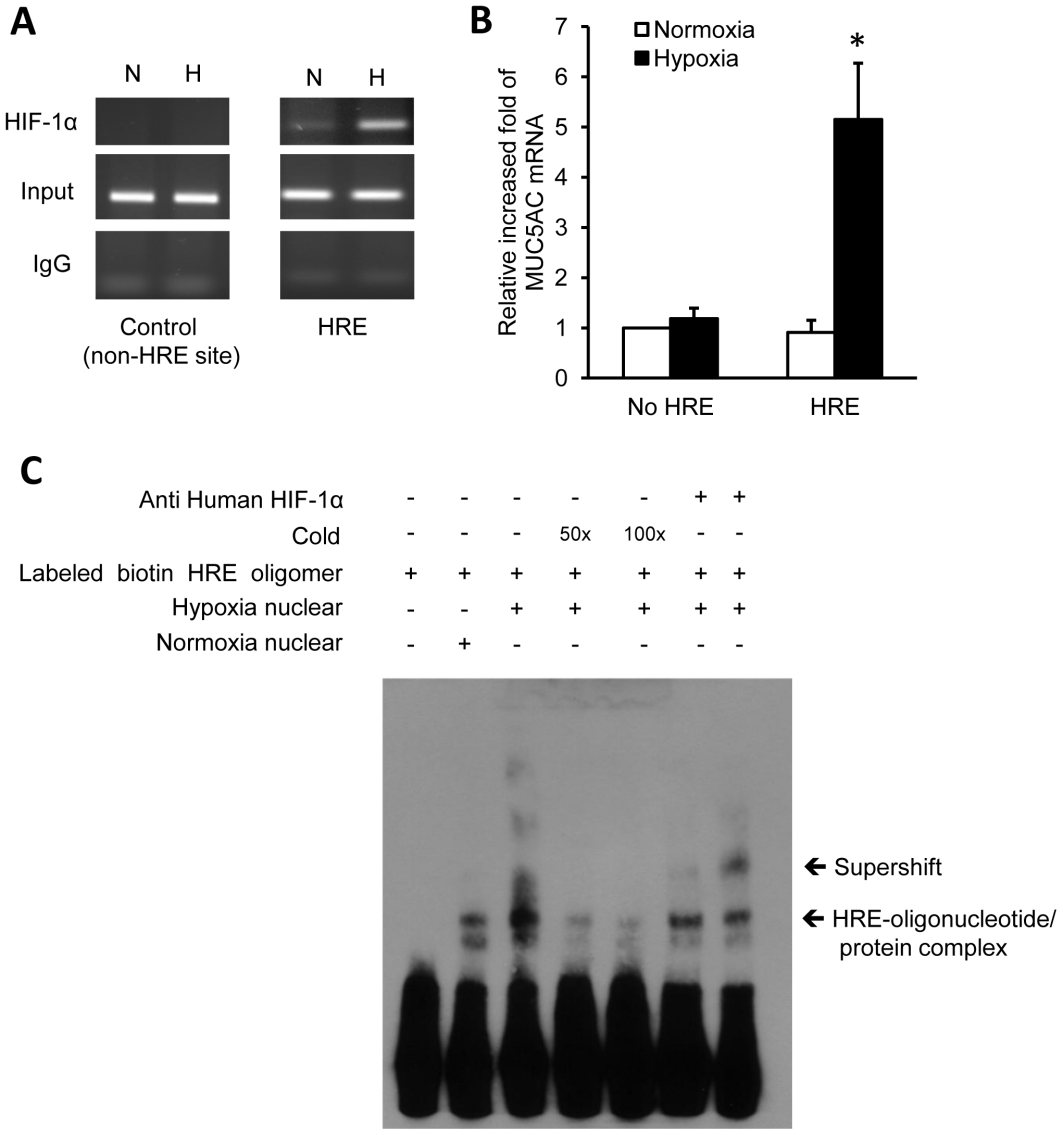
The HRE is required for anoxia-induced MUC5AC transcription. (**A**) Chromatin immunoprecipitation (ChIP) assay. NCI-H292 cells were transfected with vectors encoding HIF-1α-binding flanking region of the MUC5AC promoter (HRE site), or a region that does not contain an HRE region (non-HRE site; negative control). The cells were treated under normoxic or anoxic conditions for 6 h and subjected to immunoprecipitation with HIF-1α antibody or control IgG. (**B**) The MUC5AC mRNA transcript was analyzed by real-time PCR. The binding to HRE under anoxic conditions significantly increased the expression of MUC5AC expression compared to non-HRE region, or HRE region under anoxic conditions (n = 3, *p<0.05). (**C**) EMSA to determine HIF-1α binding to the HRE region of the MUC5AC promoter in response to anoxia. The activity with HIF-1α-specific HRE-containing oligonucleotides is increased remarkably in response to anoxia. The EMSA band of interest was found to be selectively inhibited by specific HRE-containing competitor oligonucleotide, and it was super-shifted by anti-HIF-1α antibody. Data is shown as mean ± standard deviation. (*N; normoxia, A;* anoxia).

To determine the DNA binding activity of hypoxia-induced HIF-1α to the MUC5AC promoter, we performed chromatin immunoprecipitation (ChIP) assays using nuclear extracts of hypoxia-treated NCI-H292 cells (Fig. 5A and C).

The binding of HIF-1α to the HRE region of the MUC5AC promoter during hypoxia was confirmed by ChIP assay. The MUC5AC mRNA transcript was analyzed by real-time PCR (Fig. 5B). The binding to HRE under anoxic conditions significantly increased MUC5AC mRNA compared to the non-HRE or HRE region (p<0.05).

To confirm the DNA binding activity of hypoxia-induced HIF-1α, we performed an EMSA using nuclear extracts from NCI-H292 cells after normoxic or anoxic stimulation (Fig. 5C). The activities of HIF-1α specific HRE-oligonucleotides increased remarkably in response to anoxia. To distinguish any specific HRE-binding complexes, competition and super-shift analysis were performed using 50- or 100-fold excesses of nonradiolabeled (cold) HRE oligonucleotide and anti-HIF-1α antibody, respectively. The specific band was found to be selectively inhibited by the specific HRE competitor, and it was super-shifted by anti-HIF-1α antibody. These results show that HRE in the regulatory region of the MUC5AC promoter is important for the up-regulation of MUC5AC transcriptional activity by hypoxia.

### Overexpression of HIF-1 and MUC5AC in hypoxic sinonasal mucosa

**Figure 6:**
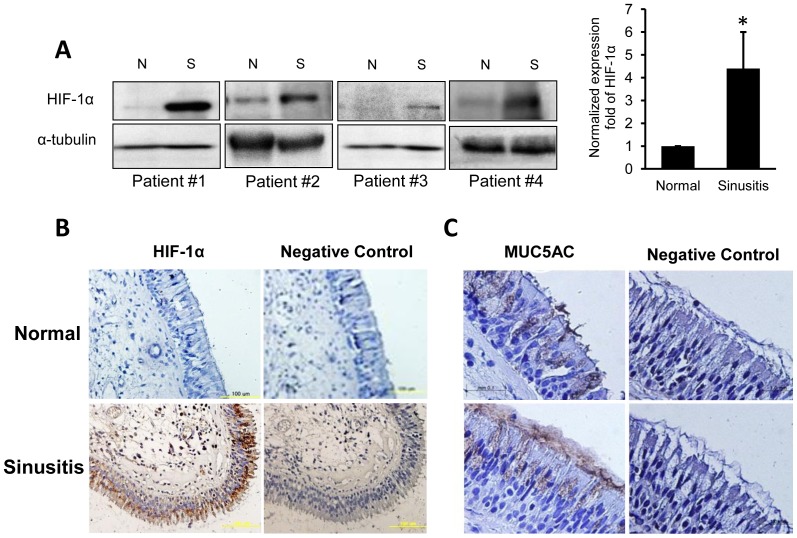
Sinus mucosa from chronic sinusitis patients shows high levels of HIF-1α and MUC5AC expression. (**A**) The expression of HIF-1α in the control and sinus mucosa from four patients with sinusitis (western blot analysis). The increase of HIF-1α expression in sinusitis was 4.4-fold greater than in control tissue (n = 4; *p<0.05). (**B**) Immunohistochemistry with anti-HIF-1α antibody in normal (left) and sinusitis (right) tissues. High HIF-1α protein expression is indicated by the strong antibody reactivity in the epithelium of sinus mucosa from a sinusitis patient. (**C**) Immunohistochemistry with anti-MUC5AC antibody in normal (upper) and sinusitis (lower) tissues. Pronounced MUC5AC expression is seen in the epithelium from the sinusitis patient. Data is shown as mean ± standard deviation. *(N; normal, S; sinusitis)*.

The expression of HIF-1α was also determined in sinusitis patients. The mucosa was harvested from both diseased and control sinus from four individual patients, and their HIF-1α expressison levels were compared by Western blot (Fig. 6A). Normalized mean-fold of HIF-1α expression level was 4.4-times greater than control (n = 4; *p<0.05). Strong expression of HIF-1α was also noted in immunohistochemical analysis of epithelium from sinusitis (Fig. 6B). We also confirmed MUC5AC expression in human tissue with immunohistochemical staining, and it was strongly expressed in epithelium from sinusitis compared to control tissue (Fig. 6C).

## Discussion

This study was performed based on the presumption that conditions of hypoxia within the sinus can directly induce mucus hypersecretion, which is one of the striking features of sinusitis. The sinus mucosa is lined with ciliated columnar epithelium interspersed with goblet cells. The cilia of epithelial cells have an important role in sweeping mucus toward the ostium, an outflow tract of sinus, and maintaining a healthy microenvironment within the sinus cavity. Normal mucociliary transport is critical for the optimal maintenance of the airway mucosa, and a decrease in the activity has been observed in rhinosinusitis. The decrease in mucociliary clearance and mucostasis can be caused by alteration of mucus viscoelasticity or any direct toxic effects on cilia [23]. Hypoxia is another causal factor associated with sinusitis, as it is responsible for various inflammatory cytokines and mediators, including vascular endothelial growth factor, inducible nitric oxide synthase, matrix metalloproteinases, as well as transforming growth factor-β [24]. The obstruction of the ostium reduces oxygen tension within the sinus and causes sinusitis [8]. Goblet cell or mucous metaplasia is one of major histopathologic changes in chronic rhinosinusitis [1], and activation of HIF-1α mediates the associated changes via the extracellular signal-regulated kinase 1/2 signaling pathway [25]. Although hypoxia is known as a potent stimulant of inflammation [9] and tissue remodeling that contributes to sinus diseases, the effect of hypoxia on mucin production and its mechanism in airway epithelium has not been clearly understood.

This study has demonstrated that the MUC5AC gene is directly induced by hypoxia in normal human nasal epithelial cells (Fig. 1). The transcriptional activation of MUC5AC occurred after exposure to hypoxic conditions. One possible mechanism by which hypoxia regulates MUC5AC gene expression is through the transcription factor, HIF-1. This factor is a heterodimeric protein composed of an oxygen-regulated α subunit and an oxygen-independent β subunit [26]. The α subunits are continuously transcribed and translated. Under normoxic conditions, two prolines in the α subunit are hydroxylated, which enables the protein to be ubiquinated by the von Hippel-Lindau tumor suppressor and degraded in the 26S proteosome [27].

When oxygen levels decrease during hypoxia, α and β subunits of HIF-1 heterodimerize and bind to specific HRE elements in promoters of many genes, thereby inducing transcription. In this study, although mRNA expression of HIF-1α was not influenced by anoxic stimulation (Fig. 2A), the protein level of HIF-1α was increased with time under anoxic conditions (Fig. 2B). This effect suggested a post-translational stabilization of HIF-1α gene.

We found that a specific MUC5AC promoter region contains a putative HRE. The HRE of the MUC5AC promoter exhibits a core sequence ACGTG that is similar to the sequences found in the promoters of transferrin and lactate dehydrogenase. To evaluate the role of HIF-1 as assessed by its over-expression, HIF-1α expression vectors were transfected into NCI-H292 cells. The MUC5AC promoter activity was enhanced by increased expression of HIF-1α, even under normoxic conditions. However, compared to the increased amount of HIF-1α protein (Fig. 4), the increased MUC5AC mRNA levels in the cells with over-expression of HIF-1α did not appear to be sufficient. This may suggest that additional over-expression of HIF-1β could contribute to a higher activity of the MUC5AC promoter, as observed in other studies in which cotransfection of HIF-1α and HIF-1β resulted in higher activity of transferrin receptor promoter than HIF-1α alone [23]. These results suggest that the MUC5AC gene contains an enhancer regulated by HIF-1α. Moreover, our studies demonstrated that the wild-type (not HRE-mutated) MUC5AC promoter conferred inducibility to the luciferase reporter gene under hypoxic conditions in NCI-H292 cells (Fig. 4). However, although the relative difference in activity between cells incubated under hypoxic versus normoxic conditions was significant, it was not extremely great. This indicates that there may be yet another mechanism that is involved in activating the MUC5AC gene by mediation through the wild-type HRE, other than the mechanism involving HIF-1α.

Because the wild-type HRE-mutated MUC5AC promoter attenuates the reporter activity, the wild-type HRE of the MUC5AC promoter might be a strong candidate for a functional HRE. This conclusion is also supported by ChiP assay results for HRE (Fig. 5A) and by EMSA super-shift data (Fig. 5B).

Our study reveals that hypoxia contributes to sinus disease by the alteration of MUC5AC production, via the activation of HIF-1α. In the specimens from sinusitis patients, over-expression of HIF-1α and MUC5AC was observed by immunohistochemistry (Fig. 6). MUC5AC is one of the major secreted mucins in sinusitis. Considering that a marked increase of MUC5AC and MUC5B secretion has been reported in sinus disease, and MUC5AC expression was found to be higher compared to MUC5B in sinus [28], the role of hypoxic stress to mucosal epithelium in the pathophysiology of sinusitis could be an area where greater attention should be paid clinically. The measured oxygen tension of sinus in sinusitis patients varied from 0.7% to 21.6% according to disease chronicity or degree of ostial obstruction [29]. In this study, the condition of hypoxia was close to anoxic state. The hypoxic condition of 1.5% O_2_ was tested for 6, 12, 16, and 24 h of exposure. Unlike the results under anoxic condition, MUC5AC mRNA under hypoxia condition (1.5% O_2_) was not induced until 16 h, but increased at 24 h of time point. But, the increased level was modest, about 1.6 fold of increase (Fig. S1). Based on the results of our additional experiments, hypoxic condition looks have less effect on MUC5AC compared to anoxic state, although HIF-1α was induced by hypoxia. Hypoxic condition needs a longer time (at least 24 h of exposure) to elevate MUC5AC mRNA. An anoxic condition is physiologically not relevant to chronic sinusitis, but the sinus mucosa chronically exposed to hypoxic condition may have an influence on MUC5AC secretion. Therefore, in the management of upper airway diseases, especially rhinosinusitis, the mechanical or pharmacological restoration of ventilation to lessen hypoxia could be beneficial in reducing the pathological hypersecretion of mucus.

## Methods

### Cell culture

This study was approved by the Institutional Review Board of Yonsei University College of Medicine. All participants provided their written consent to participate in this study.

Passage-2 normal human nasal epithelial (NHNE) cells were prepared as described previously [30]. Passage-2 NHNE cells (1×10^5^ cells) were seeded in 0.5 ml of culture medium on 24.5-mm, 0.45-µm pore size, Transwell-clear (Costar Co., Cambridge, MA, USA) culture inserts. Cells were cultured in a 1∶1 mixture of bronchial epithelial growth medium: Dulbecco's modified Eagle's medium containing all supplements [31]. The cells were grown submerged for the first 9 days; an air-liquid interface was created on day 9 by removing the apical medium and restricting the culture feeding to the basal compartment. The air-liquid process was accomplished in a 37°C humidified cell incubator infused with 5% carbon dioxide of air and this is essential to differentiate NHNE cells into ciliated respiratory epithelial cells, which mimic real airway epithelium. The cultured epithelial cells were treated with hypoxia on day 16 of culture.

The human lung mucoepidermoid carcinoma cell line (NCI-H292) was purchased from the American Type Culture Collection (CRL-1838) (Manassas, VA, USA) and cultured in RPMI 1640 medium (Invitrogen, Carlsbad, CA, USA) supplemented with 10% (v/v) fetal bovine serum in the presence of penicillin/streptomycin at 37°C in a humidified chamber with 5% CO_2_. Contrary to NHNE cells, culture of NCI-H292 cells does not need a process of air-liquid interface. For serum deprivation, confluent cells were washed twice with phosphate-buffered saline and recultured in RPMI with 0.2% (v/v) fetal bovine serum. Hypoxic condition, which was actually anoxia, were produced in a Forma 1029 anaerobic chamber (Thermo Fisher Scientific; Waltham, MA, USA) perfused with continuous flow of a mixture of 95% N_2_/5% CO_2_ gas. Monitoring the O_2_ tension to identify anoxic state and potential air leak was performed by BBL GasPak Disposable Anaerobic Indicator (Becton Dickson company, MD, USA) which changes color when O_2_ tension reaches 0.5% as performed by Zhu et al [32]. Cell harvest was performed under normoxia quickly immediately after anoxic treatment and it was enough to observe HIF-1α before its degradation. We also tested the expression of HIF-1α in cells those had been lysed in anoxic incubator with same 95% N_2_/5% CO_2_ atmosphere. The HIF-1 expression levels under two experimental conditions were compared and we could not observe any difference between methods.

### RNA isolation and RT-PCR

Total RNA was isolated from NHNE cells or the NCI-H292 lung cancer cell line using TRIzol (Invitrogen; Carlsbad, CA, USA). cDNA was synthesized with random hexamers (PerkinElmer Life Sciences, Waltham, MA, USA) using the Moloney murine leukemia virus reverse transcriptase (PerkinElmer Life Sciences; Waltham, MA, USA). Oligonucleotide primers for PCR were designed based on the GenBank sequences listed in Table 1. The PCR conditions for MUC5AC involved 35 cycles of: denaturation at 94°C for 30 s, annealing at 60°C for 30 s, followed by polymerization at 72°C for 30 s. PCR parameters for HIF-1α consisted of 24 cycles of: denaturation at 94°C for 30 s, annealing at 55°C for 30 s, and polymerization at 72°C for 30 s. PCR parameters for β2-microglobulin contained 24 cycles of: denaturation at 94°C for 30 s, annealing at 55°C for 30 s, and polymerization at 72°C for 30 s. The PCR products were separated by electrophoresis in a 2% (w/v) agarose gel and visualized by ethidium bromide staining under transillumination.

**Table 1:** Primers used for PCR.

Gene name	Primer sequences	Product size
MUC5AC (RT-PCR) AJ0014029	Forward 5′-CGA CAA CTA CTT CTG CGG TGC-3′	337 bp
	Reverse 5′-GCA CTC ATC CTT CCT GTC GTT-3′	
MUC5AC (Real-time PCR)	Forward 5′-CAG CCA CGT CCC CTT CAA TA-3	66 bp
	Reverse 5′-ACC GCA TTT GGG CAT CC-3′	
β2-Microglobulin (RT-PCR) NM004048	Forward 5′-TCG CGC TAC TCT CTC TTT CTG G-3′	334 bp
	Reverse 5′-GCT TAC ATC TCT CGA TCC CAC TTA A-3′	
β2-Microglobulin (Real-time PCR)	Forward 5′-CGC TCC GTG GCC TTA GC-3′	67 bp
	Reverse 5′-GAG TAC GCT GGA TAG CCT CCA-3′	
HIF-1α (RT-PCR) NM181054	Forward 5′-GCA GCC AGA TCT CGG CGA AG-3′	319 bp or 320 bp
	Reverse 5′-CTG TGT CCA GTT AGT TCA AAC TG-3′	

RT-PCR; reverse transcriptase polymerase chain reaction.

### Real-time quantitative PCR

Real-time quantitative PCR analysis of MUC5AC gene expression was carried out with an Applied Biosystems 7300 Fast Real-Time PCR system, using SYBR Green PCR Core Reagents (Applied Biosystems; Foster City, CA, USA). Reactions were performed in a total volume of 20 µl, which included 10 µl 2× SYBR Green PCR Master Mix, 300 nM of each primer, and 1 µl previously reverse-transcribed cDNA template. Primers used are listed in Table 1. The thermocycler parameters were 50°C for 2 min, 95°C for 20 s, followed by 40 cycles of 95°C for 15 s and 60°C for 30 s. All reactions were performed in triplicate. The relative quantity of MUC5AC mRNA was obtained using the comparative cycle threshold method and normalized using β2-microglobulin as an endogenous control.

### Western blot analysis

After anoxic treatment, NHNE or NCI-H292 cells were lysed with 2× lysis buffer (250 mM Tris-HCl at pH 6.5, 2% w/v sodium dodecyl sulfate (SDS), 4% v/v mercaptoethanol, 0.02% w/v bromphenol blue, 10% v/v glycerol). Equal amounts of whole cell lysates were resolved by SDS-polyacrylamide gel electrophoresis and transferred to a polyvinylidene difluoride membrane (Millipore; Bedford, MA, USA). Membranes were blocked with 5% (w/v) skim milk in Tris-buffered saline (50 mM Tris-HCl at pH 7.5 and 150 mM NaCl) for 2 h at room temperature. The blot was incubated overnight with primary antibody (BD Bioscience; San Jose, CA, USA) in 0.5% v/v Tween 20 in Tris-buffered saline (TTBS). After washing with TTBS, the blot was further incubated for 45 min at room temperature with anti-mouse antibody (Cell Signaling; Danvers, MA, USA) in TTBS and visualized by the ECL kit (Amersham; Little Chalfont, Buckinghamshire, UK).

### Small interfering RNA transfection

The role of HIF-1 in mediating MUC5AC expression under hypoxic conditions was determined by transfection of HIF-1α small-interfering RNA (siHIF-1α) to silence the HIF-1α gene. The HIF-1α siRNA oligonucleotides (Stealth siRNA) were synthesized by Invitrogen (Invitrogen, Carlsbad, CA, USA). We screened HIF-1 mRNA (GenBank NM181054) and selected potential siRNA sequences with high values of knock-down probability. The siRNA sequences selected were 5′-GUG GUU GGA UCU AAC ACU A-3′. Stealth RNAi negative control duplex (Medium GC, Invitrogen) was used as an siRNA negative control. siRNA transfection into NCI-H292 cells was carried out with Lipofectamine 2000 (Invitrogen) according to the manufacturer's instructions. Ten picomole of each siRNA and 2 µl Lipofectamine were mixed with RPMI without serum and antibiotics and transfection was performed into NCI-H292 cells growing in six-well culture plates when the cultures reached 30–50% confluence. This procedure did not affect cell viability. The same procedure was performed with control siRNA.

### Quantification of secreted MUC5AC

To analyze the production of MUC5AC, accumulated apical secretion fluid was collected and subjected to dot blot analysis. During culture of NHNE cells, basolateral side is submerged into culture medium, but apical side is exposed to air. The cells secreted mucus onto their surface and this can be obtained after normoxic or anoxic condition. Briefly, diluted apical secretion fluid was applied to nitrocellulose membranes, which were incubated with anti-human MUC5AC antibody, followed by reaction with horseradish peroxidase-conjugated goat anti-mouse IgG. The signal was detected by chemiluminescence using the ECL kit.

### Chromatin immunoprecipitation assay

For chromatin immunoprecipitation (Chip) assays, approximately 2∼3×10^9^ NCI-H292 cells in 150-mm dishes were treated with PBS containing 1% (w/v) formaldehyde for 10 min, washed twice with PBS and fixed with 125 mM glycine at room temperature for 5 min. The cells were rinsed twice with PBS and resuspended in 1 ml of solution A (10 mM HEPES at pH 6.5, 0.25% v/v Triton X-100, 10 mM EDTA, 0.5 mM EGTA) by pipetting. After centrifugation at 3,000 rpm for 4 min, the pellets were resuspended in solution B (10 mM HEPES at pH 6.5, 200 mM NaCl, 1 mM EDTA, 0.5 mM EGTA) containing protease inhibitors by vigorous pipetting to extract nuclear proteins. After centrifugation at 4,000 rpm for 5 min, the nuclear pellets were resuspended in immunoprecipitation buffer (10 mM EDTA, 50 mM Tris-HCl at pH 8.1, 1% w/v SDS, 0.5% w/v Empigen BB) containing protease inhibitors and sonicated to break the chromatin into fragments with an average length of 0.5–1 kb. The cells were treated under normoxic or hypoxic conditions. The following antibodies were used in the assay: 2 mg of anti-HIF-1α antibody and negative control IgG (2 mg, rabbit) that either amplified the HIF-1α-binding flanking region in the MUC5AC promoter (HRE site) or a further upstream region that does not contain a HIF-1α-binding site (non-HRE site). The MUC5AC mRNA transcript was determined by real-time PCR.

### Transient transfection and luciferase assay

NCI-H292 cells were transiently transfected with the following plasmids: 1) for gain-of-function study of HIF-1α, with pCMV-basic and pCMV-HIF-1α plasmids (generously given by Dr. Koh EM, SMC, Seoul, Korea); 2) for MUC5AC promoter luciferase assay, with pGL3-basic, pGL3-MUC5AC promoter (−1400/+4), and pGL3-mutated MUC5AC promoter (−1400/+4) plasmids using the FuGENE 6 transfection reagent (Roche Applied Science; Basel, Switzerland) according to the manufacturer's instructions. After transfection of reporter plasmids, transfected cells were incubated under defined conditions for 6 h and assayed for luciferase activity, using a luciferase assay system (Promega; Madison, WI, USA), according to the manufacturer's instructions. β-galactosidase activity was also assayed to standardize the transfection efficiency of each sample. Mutation of the putative regulatory elements that start from −65 bp upstream of the MUC5AC gene was generated by mutagenic PCR (5′-ACGTG-3′ mutated to 5′-AAATG-3′; positions −65 to −61).

### Electrophoretic mobility shift assay

The binding activity of HIF-1α and HRE on the MUC5AC promoter was detected by electrophoretic mobility shift assay (EMSA). The single-stranded oligonucleotide sequence of 5′-ccc acc **cac gtg** aag cac g-3′, 3′-cgt gct tc**a cgt g**gg tgg g-5′, which corresponds to the HIF-1α binding site, was end labeled with biotin (100 pmol; Bioneer, Daejeon, Korea). Cells were resuspended in cell homogenization buffer containing 0.05% (v/v) nonidet P-40 and homogenized. Next, nuclei were pelleted and resuspended in cell resuspension buffer (40 mM HEPES at pH 7.9, 0.4 M KCl, 1 mM dithiothreitol, 10% v/v glycerol, 0.1 mM phenlmethylsulfonylfluoride, 0.1% w/v aprotinin, and 0.3 M NaCl). The nuclear extract was centrifuged at 14,000 rpm for 10 min at 4°C, and the supernatant was aliquoted and stored at −70°C. Nuclear extract (5 µg) was incubated at room temperature for 20 min with biotin-labeled HRE oligonucleotide in Chemiluminescent Nucleic Acid Detection Kit (Thermo; Rockford, IL, USA). The oligo-nuclear-extract complex was separated by electrophoresis through 6% (w/v) nondenaturing polyacrylamide gels in 0.5× Tris borate-EDTA buffer. The gel was transferred to Biodyne B precut modified nylon membranes (Thermo). Blotting was done with the Chemiluminescent Nucleic Acid Detection Kit (Thermo).

### Immunohistochemical staining for MUC5AC and HIF-1α

Diseased mucosal samples were obtained from the maxillary sinus of patients with sinusitis, which had been confirmed by computed tomography (CT). Control tissues were obtained from normal-appearing ethmoidal mucosa of the same subjects when performing endoscopic sinus surgery. The tissues were fixed with 10% (w/v) formaldehyde solution for 24 h and dehydrated and embedded in paraffin. Paraffin blocks were sectioned into 4-µm-thick slices and fixed. After deparaffinization and rehydration, slides were incubated in antigen retrieval solution (Tris-EDTA, pH 9.0) for 20 min at 90–95°C. To block endogenous peroxidase, slides were treated with 0.3% (v/v) H_2_0_2_ for 15 min at room temperature. Slides were blocked with 10% (v/v) normal serum with 1% (w/v) bovine serum albumin in TBS for 2 h at room temperature and incubated overnight at 4°C with a monoclonal mouse antibody against human MUC5AC (1∶100; Jackson Immune Research; Sacramento, CA, USA) and HIF-1α (BD Bioscience; San Jose, CA, USA). The slides were incubated with horseradish peroxidase-conjugated goat anti-mouse IgG (1∶200; Jackson Immune Research) in antibody diluent solution (DAKO; Glostrup, Denmark) at room temperature and counterstained with hematoxylin (Merck, Darmstadt, Germany).

### Statistical analysis

Data are presented as mean values ± standard deviation, calculated from at least three independent experiments. Statistical differences were analyzed by one-way analysis of variance (ANOVA) with Dunnett's multiple comparison post-hoc test, two-way ANOVA test or Wilcoxon-Mann-Whitney tests, and a p-value of less than 0.05 was considered to be statistically significant.

## Conclusions

Mucus hypersecretion is one of major clinical features of rhinosinusitis, and hypoxic conditions in the sinus have been suggested as its pathophysiology. Our study has revealed that the production of MUC5AC is increased through activation of HIF-1α under hypoxic stimulation, and the HRE promoter region of MUC5AC seems to be critical to regulate MUC5AC production. Therefore, increasing the oxygen tension in the sinus may result in improvement in sinusitis by reducing MUC5AC production, and modulation of HIF-1α activity should be considered as a new target for the treatment of sinusitis.

## Supporting Information

Figure S1 **Hypoxia induces MUC5AC mRNA expression in NHNE cells.** The level of MUC5AC mRNA under hypoxia condition (1.5% O_2_) at 12 or 16 h was not induced, but showed modestly increase of 1.6 fold at 24 h of time point.
(TIFF)
