# Supplementary figures and images for: Hypoxia-Mediated Mechanism of MUC5AC Production in Human Nasal Epithelia and Its Implication in Rhinosinusitis
Source: PLoS One. 2014 May 19;9(5):e98136. doi: 10.1371/journal.pone.0098136 (PMC4026485; doi:10.1371/journal.pone.0098136)

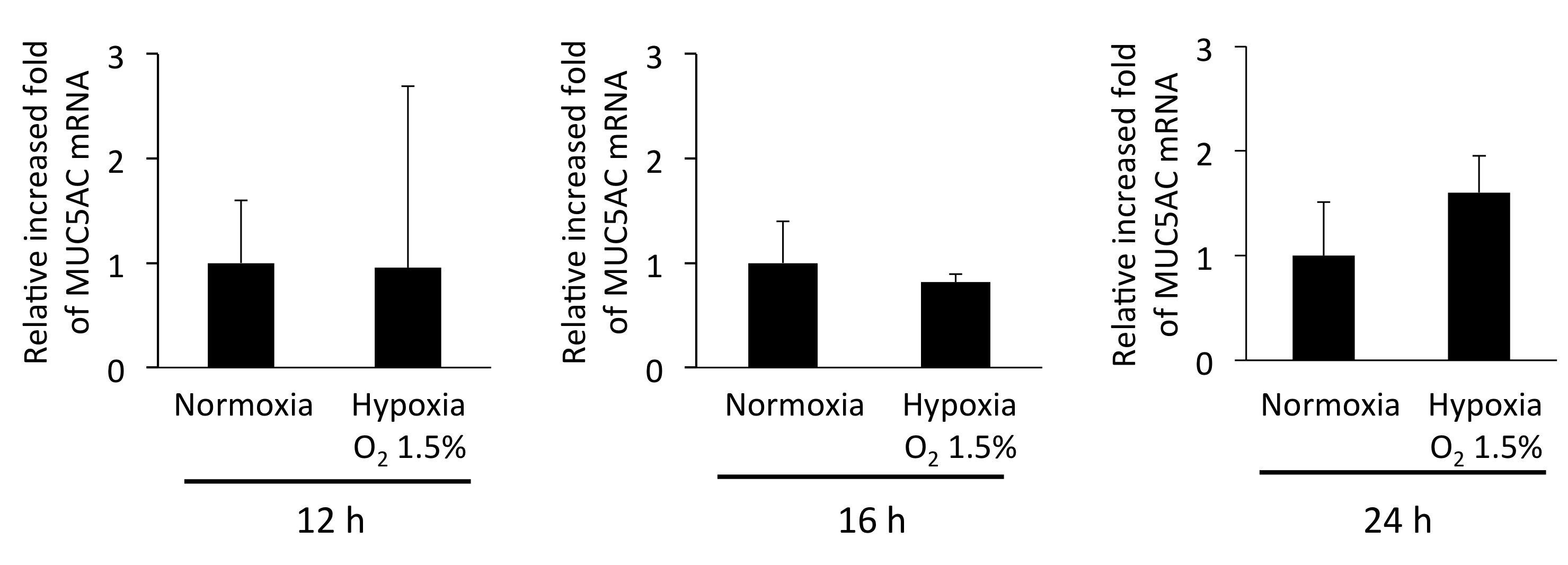

Supplement: Figure S1 — Hypoxia induces MUC5AC mRNA expression in NHNE cells. The level of MUC5AC mRNA under hypoxia condition (1.5% O2) at 12 or 16 h was not induced, but showed modestly increase of 1.6 fold at 24 h of time point. (TIFF) [file pone.0098136.s001.tiff]
